# An Additive Effect of Oral N-Acetyl Cysteine on Eradication of *Helicobacter pylori*

**DOI:** 10.1155/2015/540271

**Published:** 2015-09-02

**Authors:** Seyed Mohammad-Taghi Hamidian, Najmeh-sadat Aletaha, Reza Taslimi, Mohammad Montazeri

**Affiliations:** ^1^Division of Gastroenterology, Department of Internal Medicine, Babol University of Medical Sciences, Babol 47176 41367, Iran; ^2^Division of Gastroenterology, Department of Internal Medicine, Tehran University of Medical Sciences, Tehran, Iran; ^3^Young Researchers Club, Islamic Azad University, Babol Branch, Babol, Iran

## Abstract

*Background. Helicobacter pylori* is highly adapted to the gastric environment where it lives within or beneath the gastric mucous layer. The aim of this study was to evaluate whether the addition of N-acetyl cysteine to the treatment regimen of *H. pylori* infection would affect eradication rates of the disease. *Methods.* A total of 79 *H. pylori* positive patients were randomized to two therapeutic groups. Both groups received a 14-day course of three-drug regimen including amoxicillin/clarithromycin/omeprazole. Experimental group (38 subjects) received NAC, and control group (41 subjects) received placebo, besides three-drug regimen. *H. pylori* eradication was evaluated by urea breath test at least 4 weeks after the cessation of therapy. *Results.* The rate of *H. pylori* eradication was 72.9% and 60.9% in experimental and control groups, respectively (*P* = 0.005). By logistic regression modeling, female gender (OR 3.68, 95% CI: 1.06–5.79; *P* = 0.040) and treatment including NAC (OR 1.88, 95% CI: 0.68–3.15; *P* = 0.021) were independent factors associated with *H. pylori* eradication. *Conclusion.* The results of the present study show that NAC has an additive effect on the eradication rates of *H. pylori* obtained with three-drug regimen and appears to be a promising means of eradicating *H. pylori* infection.

## 1. Introduction

*Helicobacter pylori* is a Gram-negative microaerophilic spiral-shaped bacterium that is the most common chronic bacterial infection in humans with a incidence of up to 70% in developing populations [1]. This bacterium has a key role in the pathogenesis of chronic active gastritis, peptic ulcers, gastric cancers, and gastric mucosa associated lymphoid tissue lymphomas [2]. For* H. pylori* infection, triple therapies have been the first-line treatment choice since the introduction of the initial published guidelines that involve two antibiotics and a proton pump inhibitor (PPI) [3]. Though recent researches have showed that the use of the standard triple therapies worldwide decreases rates of* H. pylori* eradication, consequently, new therapies that are effective, safe, and without significant side effects are required [4, 5].

N-Acetylcysteine (NAC), which is the acetylated variant of the amino acid L-cysteine, is both a thiol-containing antioxidant and a mucolytic agent [6]. It impacts based on breaking the disulphide bridges of the high-molecular-weight mucus glycoproteins, resulting in decreased viscosity. This impact clarifies the NAC action in the treatment of pulmonary conditions such as chronic bronchitis and cystic fibrosis for dispersing the characteristic heavy secretion of mucus [6, 7]. In addition, NAC may prepare scavenge reactive oxygen species (ROS) and SH-groups itself. NAC not only has antioxidant function but also has several other mechanisms of action, including inhibition of neutrophil activation, vasodilation, and reduced microbial attachment [8, 9].

A limited number of studies have assessed whether lowering the gastric mucus viscosity might impact the treatment result of* H. pylori* infection and proposed that if NAC is applied as an adjunct to standard therapy in* H. pylori* eradication, it may have an additive influence [8–11]. The purpose of the current study was to assess the effect of adding oral N-acetylcysteine to the* H. pylori* infection treatment regimen on eradication rates of* H. pylori* in subjects who require treatment against this disease.

## 2. Methods

### 2.1. Study Design and Patients

This study had a double-blind, randomized placebo-controlled design and was performed between June 2012 and July 2013 at Endoscopy Ward of Imam Khomeini Hospital of Tehran, Iran. All patients with peptic-like epigastric pain and dyspeptic symptoms, referred to the endoscopy unit and scheduled for upper GI endoscopy, were recruited for this prospective study. They were eligible to enter the study if they had gastric* H. pylori* infection. The diagnosis of* H. pylori* infection was made based on positive rapid urease test.

Exclusion criteria for patients' recruitment were recent use of antibiotics, bismuth salts, or proton pump inhibitors (PPI), chronic use of nonsteroidal anti-inflammatory drugs or corticosteroids, gastric outlet obstruction, pregnancy, renal failure, gastric malignancy, and prior gastric surgery. The study was approved by the Ethical Committee of Tehran University of Medical Sciences, and informed consents were obtained from all the participating patients prior to the treatments.

Information on smoking and drinking habits were obtained from a questionnaire filled by all subjects at the beginning of the study. Patients were defined as smokers if they smoked 5 or more cigarettes per day and as drinkers if they consumed 25 g or more alcohol per week.

### 2.2. Eradication Therapy

Patients were randomized using a computerized random-number generator to one of two therapeutic groups receiving a 14-day course of oral treatment as follows:

- The experimental group was given amoxicillin (capsules, 1000 mg) bid (with breakfast and dinner), clarithromycin (capsules, 500 mg) bid (with breakfast and dinner), and omeprazole (capsules, 20 mg) bid (before breakfast and dinner), plus NAC 600 mg bid.
- The control group was given amoxicillin/clarithromycin/omeprazole the same as experimental group, plus placebo.

In patients without peptic ulcer, omeprazole 20 mg once daily was continued for 2 weeks. But patients with peptic ulcer were treated for 4–6 additional weeks. The drugs were taken 30 minutes before meals with half a glass of water.

### 2.3. Follow-Up Procedures

Patients were asked to return at least 4 weeks after the cessation of therapy to assess eradication. Urea breath test (UBT) was performed in both groups to confirm* H. pylori* eradication. The carbon-13 urea breath test (^13^C-UBT) was performed using 100 mg of ^13^C urea and a single post-urea breath sample at 30 min. The procedure was modified from the European standard protocol for detection of* H. pylori* [12].

### 2.4. Statistical Analysis

Statistical analysis was performed using the SPSS software for Windows (version 18.0, SPSS Inc., Chicago, IL, USA). Student's *t*-test was used to compare quantitative variables between the groups. Differences between qualitative variables were evaluated with the chi-square and Fisher's exact test. Logistic regression was used to estimate risk factors in relation to eradication therapy for* H. pylori*. *P* values of less than 0.05 were considered to indicate statistical significance.

## 3. Results

A total of 79 subjects were enrolled in the study; 38% were males and 62% were females. The mean age was 42.16 ± 12.39 years. Among these patients, 41 and 38 patients were randomly placed in control and experimental group, respectively. In Table 1, the demographic information for these subjects is summarized with respect to two groups. There were no statistical differences between the baseline characteristics of these two groups.

The* H. pylori* eradication rate in all patients was 65.8 percent (52 patients). The infection was eradicated in 72.9% of patients in the experimental group (27 of 38) and in 60.9% of patients in the control group (25 of 41). A significant difference was observed between the control group and the experimental group (*P* = 0.005). The eradication rate in women was significantly higher than that in men (73.5% versus 53.5%, *P* = 0.037).

In Table 2, the* H. pylori* eradication rate is shown based on demographics and clinical characteristics in two groups. The eradication rate was higher in experimental group for both females and males (*P* = 0.044 and *P* = 0.037, resp.). Moreover, in experimental group for subjects with peptic ulcer, the* H. pylori* eradication was significantly higher (73.3% versus 58.3%, *P* = 0.048). No significant differences were observed in* H. pylori* eradication in drinker and smoker subjects.

By using logistic regression modeling, treatment including NAC (OR 1.88, 95% CI: 0.68–3.15; *P* = 0.021) and female gender (OR 3.68, 95% CI: 1.06–5.79; *P* = 0.040) were independent factors associated with eradication of* H. pylori*. Conversely, it was found that smoking (OR 0.28, 95% CI: 0.04–1.96; *P* = 0.038) is independent factor against* H. pylori* eradication. In multivariate analysis, other factors had no significant impact on the eradication efficacy of* H. pylori* (Table 3).

## 4. Discussion

The results of the present study indicate that NAC has an additive effect on the eradication rates of* H. pylori* obtained with triple amoxicillin, clarithromycin, and omeprazole and seems to be a hopeful means of eradicating* H. pylori* infection.

Moreover, our result is confirmed by Karbasi et al. [8] who demonstrated that eradicating* H. pylori* infection was achieved in 70% of patients receiving NAC in addition to three-drug regimen (omeprazole/ciprofloxacin/bismuth subcitrate) compared to 60.7% patients receiving only three-drug regimen. In addition, Gurbuz et al. [9] showed that the use of NAC during* H. pylori* eradication therapy by dual therapy with lansoprazole and clarithromycin increases the rate of cure. Zala et al. [13] assessed the effect of NAC on eradication of* H. pylori* in smokers and stated that 1.2 g NAC twice a day for 10 days improved the* H. pylori* eradication in smokers treated concomitantly with omeprazole and amoxicillin. Cammarota et al. [14] reported that* H. pylori* eradication was obtained in 65% of patients who received NAC prior to a culture-guided antibiotic regime and 20% of patients who received no NAC.

It is thought that oxidative stress plays a role in* H. pylori* induced damage of mucosa.* H. pylori* cause significant infiltration of neutrophil into the gastric mucosa and neutrophils activation and macrophages with no mucosal bacterial invasion [15]. This process generates inflammatory mediators that contain reactive oxygen species (ROS) and reactive nitrogen species (RNS), which causes oxidative stress in the gastric mucosa [16, 17]. ROS agglomeration adjusts the many genes' expression and can damage DNA. This process may have a key role in gastric carcinogenesis [18].* H. pylori* not only enhances free-radical production but also is associated with antioxidant defense system and improved glutathione turnover. Oxidative stress leads to stimulation of either a reduction in antioxidant defenses or production of additional ROS performed under a variety of conditions caused by oxidative stress. The reduced levels of antioxidants and improved levels of prooxidative parameters can modify many procedures in the gastric epithelium and result in destruction of surface epithelial layers and main biological structures such as nucleic acids, proteins, lipids, and carbohydrates [19].

NAC is an antioxidant supplement containing a hydroxyl radicals scavenger, a by-product of glutathione and H_2_O_2_ [20]. It displays indirect and direct antioxidant features. Its free thiol group has the ability to interact with the ROS electrophilic groups. Furthermore, NAC applies an indirect antioxidant impact associated with its role as a precursor of glutathione. In order to overcome the harmful impacts of toxic agents, maintenance of sufficient intracellular concentrations of glutathione is necessary [21]. NAC minimizes the cellular membranes lipid peroxidation and prevents activation of nuclear factor kappa B (NF-*κ*B), a vital transcription factor of proinflammatory cytokines in gastric epithelial cells [20]. Therefore, NAC reduced the load of oxidant on the mucosa inhibiting the response of inflammatory observed during infection of* H. pylori* [20, 21].

As shown in rats, NAC changes the physicochemical features of the gastric mucus, by decreasing the gastric mucus thickness [22]. The gastric mucus layer covering gastric mucosa including mucin glycoproteins caused elasticity and viscosity of mucus. This layer is preparing protection, lubrication, and a barrier between the epithelial cells and the luminal contents. Bacteria such as* H. pylori* can survive in such rough conditions and colonize in the basal mucus gel layer due to advantages of mucus layer such as nutrition and protection from the enzymatic and acidic medium [9, 23].* H. pylori* primarily enhances production of mucin, synthesis and production of mucus, and gene expression of mucin [24]. It is obvious that the lower the mucus viscosity, the higher the* H. pylori* eradication rates. Gotoh et al. [25] demonstrated that the performance of the eradication against* H. pylori* is improved by utilizing a mucolytic agent. It appears that the mucolytic feature of NAC might prevent growth of* H. pylori* because of decrease of the mucus gel layer thickness and the mucosal surface hydrophobicity [22, 26]. Huynh et al. [11] have reported that NAC prevents the* H. pylori* growth both in vivo and in vitro. However, NAC does not change the underlying gastric inflammation caused by* H. pylori*.

In conclusion, the current study provides additional evidence that NAC has an additive effect on the* H. pylori* eradication rates obtained with triple amoxicillin, clarithromycin, and omeprazole and seems to be a hopeful means of eradicating* H. pylori* infection.

## Figures and Tables

**Table 1:** Patients demographics and clinical characteristics.

	Experimental group (%)	Control group (%)	*P* value
	*n* = 38	*n* = 41	
Age, yr (mean ± SD)	41.61 ± 10.45	42.41 ± 14.11	0.774^*∗*^
Sex			
Male	17 (44.7)	13 (31.7)	0.255^#^
Female	21 (55.3)	28 (68.3)	
Diagnosis			
Dyspepsia w/o ulcer	23 (60.5)	29 (70.7)	0.595^#^
Gastric ulcer	9 (23.7)	4 (9.8)	
Duodenal ulcer	6 (15.8)	8 (19.5)	
Cigarette smoking			
+	7 (18.4)	6 (14.6)	0.665^‡^
−	31 (81.6)	35 (85.4)	
Alcohol consumption			
+	4 (10.5)	3 (7.3)	0.705^‡^
−	34 (89.5)	38 (92.7)	

^*∗*^ *t*-test, ^#^chi-square test, and ^‡^Fisher's exact test.

**Table 2:** Comparison the rate of *H. pylori* eradication according to demographics and clinical characteristics.

	Experimental group (%)	Control group (%)	*P* value
	*n* = 38	*n* = 41	
Age (yr)			
<30	5/6 (83.3)	6/10 (60)	0.008^*∗*^
30–50	17/25 (68)	10/19 (52.6)	0.025^*∗*^
≥50	5/7 (71.4)	9/12 (75)	0.263^*∗*^
Sex			
Male	10/17 (58.8)	6/13 (46.2)	0.044^*∗*^
Female	17/21 (81)	19/28 (67.9)	0.037^*∗*^
Diagnosis			
Dyspepsia w/o ulcer	16/23 (69.6)	18/29 (62.1)	0.049^*∗*^
Gastric ulcer	7/9 (77.8)	2/4 (50)	0.013^‡^
Duodenal ulcer	4/6 (66.7)	5/8 (62.5)	0.210^‡^
Cigarette smoking			
+	4/7 (57.1)	3/6 (50)	0.617^‡^
−	23/31 (74.2)	22/35 (62.9)	0.029^*∗*^
Alcohol consumption			
+	3/4 (75)	2/3 (66.7)	0.714^‡^
−	24/34 (70.6)	23/38 (60.5)	0.036^*∗*^

^*∗*^chi-square, ^‡^Fisher's exact test.

**Table 3:** Logistic regression analysis of risk factors for eradication of *H. pylori*.

Risk factors	Odds ratio	Confidence interval	*P* value
Age >45 yr	1.67	0.78–3.58	0.186
Gender: female	3.68	1.06–5.79	0.040
Peptic ulcer	1.10	0.37–3.25	0.857
Smoking habit	0.48	0.12–1.45	0.038
Drinking habit	1.28	0.04–1.96	0.202
Treatment include NAC	1.88	0.68–3.15	0.021
